# FAM family gene prediction model reveals heterogeneity, stemness and immune microenvironment of UCEC

**DOI:** 10.3389/fmolb.2023.1200335

**Published:** 2023-05-19

**Authors:** Hao Chi, Xinrui Gao, Zhijia Xia, Wanying Yu, Xisheng Yin, Yifan Pan, Gaoge Peng, Xinrui Mao, Alexander Tobias Teichmann, Jing Zhang, Lisa Jia Tran, Tianxiao Jiang, Yunfei Liu, Guanhu Yang, Qin Wang

**Affiliations:** ^1^ Clinical Medical College, Southwest Medical University, Luzhou, China; ^2^ Department of General, Visceral, and Transplant Surgery, Ludwig-Maximilians-University Munich, Munich, Germany; ^3^ Sichuan Provincial Center for Gynecology and Breast Diseases (Gynecology), Affiliated Hospital of Southwest Medical University, Luzhou, China; ^4^ Division of Basic Biomedical Sciences, The University of South Dakota Sanford School of Medicine, Vermillion, SD, United States; ^5^ Department of Specialty Medicine, Ohio University, Athens, OH, United States

**Keywords:** UCEC, FAM family genes, tumor heterogeneity, tumor microenvironment, stemness, cancer treatment, chemotherapy

## Abstract

**Background:** Endometrial cancer (UCEC) is a highly heterogeneous gynecologic malignancy that exhibits variable prognostic outcomes and responses to immunotherapy. The Familial sequence similarity (FAM) gene family is known to contribute to the pathogenesis of various malignancies, but the extent of their involvement in UCEC has not been systematically studied. This investigation aimed to develop a robust risk profile based on FAM family genes (FFGs) to predict the prognosis and suitability for immunotherapy in UCEC patients.

**Methods:** Using the TCGA-UCEC cohort from The Cancer Genome Atlas (TCGA) database, we obtained expression profiles of FFGs from 552 UCEC and 35 normal samples, and analyzed the expression patterns and prognostic relevance of 363 FAM family genes. The UCEC samples were randomly divided into training and test sets (1:1), and univariate Cox regression analysis and Lasso Cox regression analysis were conducted to identify the differentially expressed genes (FAM13C, FAM110B, and FAM72A) that were significantly associated with prognosis. A prognostic risk scoring system was constructed based on these three gene characteristics using multivariate Cox proportional risk regression. The clinical potential and immune status of FFGs were analyzed using CiberSort, SSGSEA, and tumor immune dysfunction and rejection (TIDE) algorithms. qRT-PCR and IHC for detecting the expression levels of 3-FFGs.

**Results:** Three FFGs, namely, FAM13C, FAM110B, and FAM72A, were identified as strongly associated with the prognosis of UCEC and effective predictors of UCEC prognosis. Multivariate analysis demonstrated that the developed model was an independent predictor of UCEC, and that patients in the low-risk group had better overall survival than those in the high-risk group. The nomogram constructed from clinical characteristics and risk scores exhibited good prognostic power. Patients in the low-risk group exhibited a higher tumor mutational load (TMB) and were more likely to benefit from immunotherapy.

**Conclusion:** This study successfully developed and validated novel biomarkers based on FFGs for predicting the prognosis and immune status of UCEC patients. The identified FFGs can accurately assess the prognosis of UCEC patients and facilitate the identification of specific subgroups of patients who may benefit from personalized treatment with immunotherapy and chemotherapy.

## 1 Introduction

Uterine corpus endometrial carcinoma (UCEC) is a prevalent malignant tumor among women and accounts for a significant proportion of cancer cases worldwide (Bray et al., 2018). Despite various treatment options, including surgery, chemotherapy, radiotherapy, and hormonal therapy, the incidence and mortality rates of UCEC continue to rise each year (Soslow et al., 2019). Traditional clinicopathological staging is used to guide treatment regimens such as immunotherapy and chemotherapy, but its accuracy in predicting disease prognosis may be limited. Hence, there is an urgent need to identify novel prognostic biomarkers and molecular targets for UCEC (Zhou et al., 2020). By improving the accuracy of prognostic prediction, personalized treatment can be provided to improve patient outcomes and quality of life. Research efforts should therefore focus on identifying and characterizing new biomarkers to better understand the pathogenesis of UCEC and develop targeted treatments.

The FAM gene family comprises a group of genes that have not been fully characterized but possess similar protein sequences (Zhang et al., 2019). Several lines of research have reported on the significant participation of FAM family genes in various types of tumor pathogenesis, including proliferation, invasion, migration, and drug resistance (Bartel and Jackson, 2017; Chen et al., 2017; Li et al., 2019; Herrero et al., 2020). Furthermore, specific members of the FAM gene family have been recognized as promising therapeutic targets and/or prognostic biomarkers for the management of multiple types of cancer, such as Glioblastoma multiforme (Rahane et al., 2019), Lung adenocarcinoma (Yu et al., 2020) and Colon adenocarcinoma (Wang et al., 2020a). For example, FAM175B is mutated at a high frequency in familial breast cancer and is associated with DNA damage repair (Cava et al., 2021). These findings provide new insights into the molecular mechanisms of tumorigenesis and progression and help to reveal the pathogenesis of tumors and further investigate therapeutic approaches for tumors. Secondly, the study of FAM family genes also helps to discover new tumor markers and provides new methods and tools for early diagnosis and prognosis assessment of tumors. For example, FAM129A has decreased expression in a variety of tumors and can be used as a marker for early diagnosis and prognostic assessment of tumors (Ayesha et al., 2022). These findings provide a theoretical basis for the development of new therapeutic approaches and drugs targeting FAM family genes.

Bioinformatics techniques have made significant advancements in identifying potential biomarkers for various diseases (Lai et al., 2021; Jin et al., 2022; Shen et al., 2022; Wu et al., 2022; Chi et al., 2023a; Zhao et al., 2023a; Chi et al., 2023b; Zhao et al., 2023b; Wu et al., 2023). Prognostic models using specific gene families have also been developed through extensive research (Zhang et al., 2020; Pan et al., 2022; Wang et al., 2022). However, despite the well-established roles and mechanisms of some FAM family genes (FFGs) in various cancers, no studies have yet evaluated their prognostic and therapeutic potential in UCEC. Therefore, this investigation aimed to analyze the expression patterns of FFGs in relation to UCEC prognosis, utilizing the TCGA-UCEC dataset. By constructing a risk score, we identified three FFGs (FAM13C, FAM110B, and FAM72A) and developed a prognostic model based on FFGs. We further explored the correlation of this model with the immune microenvironment, chemotherapy, and immunotherapy. This comprehensive genomic data analysis aimed to demonstrate the potential of FAM gene family-related features in improving UCEC prognosis and patient diagnosis, providing an innovative tool for personalized treatment strategies.

## 2 Materials and methods

### 2.1 Data sources

Gene expression profiles and clinical data, including age, grade, and overall survival (OS), were obtained from the TCGA database (https://portal.gdc.cancer.gov/) for the TCGA-UCEC cohort comprising 552 UCEC samples and 35 normal samples. After excluding incomplete clinical data, 543 UCEC samples were included for subsequent analysis. The HTSeq-Fragments per kilobase million (FPKM) level 3 data for TCGA-UCEC was transformed to transcripts per million reads (TPM) using the formula TPMn = FPKMn * 106/(FPKM0 + . + FPKMm), where n represents the gene and m represents the total number of all genes. Log2-transformation was then applied to the TPM values. The cart R package was used to randomly divide the UCEC cohort into a training risk group and a test risk group in a 1:1 ratio, based on relevant clinical information.

### 2.2 Model construction

To determine the FAM family genes that may impact UCEC patient prognosis, we conducted univariate Cox regression analysis. Subsequently, we utilized the R package “glmnet” to perform Lasso-Cox regression analysis (Huang et al., 2023), which identified key genes and their corresponding regression coefficients among the FAM family genes that were significantly associated with UCEC patient prognosis (*p* < 0.05) (Friedman et al., 2010).

### 2.3 Model formulae

We generated risk scores for all patients using the model equations and then determined the optimal cut-off values using the R package “survminer”. Subsequently, all UCEC patients were categorized into high-risk and low-risk groups based on these values, and we plotted survival curves accordingly. To assess the discriminatory ability of our model, we conducted principal component analysis (PCA) using R software and calculated the C-index using the “pec” R package (Zhang et al., 2022a). Moreover, we utilized the “survivalROC” R package (Zhang et al., 2023a; Zhang et al., 2023b) to perform time-dependent ROC curve analysis and evaluate the predictive ability of genetic traits.

### 2.4 Independent prognostic analysis and nomogram construction

We conducted univariate and multivariate Cox regression analyses to assess the independent prognostic value of the risk score (Pei et al., 2023a; Pei et al., 2023b; Liu et al., 2023). Furthermore, we utilized the rms R package to generate column line plots that incorporated age, tumor stage, model gene expression, and the risk score to forecast overall survival rates at 1, 3, and 5 years for UCEC patients included in the TCGA dataset.

### 2.5 Immunity analysis of the risk signature

Several immune infiltration score measurement methods, including XCELL (Aran et al., 2017; Aran, 2020), TIMER (Chen et al., 2018; Li et al., 2020), QUANTISEQ (Finotello et al., 2019; Plattner et al., 2020), MCPCOUNT (Dienstmann et al., 2019), EPIC (Racle et al., 2017), CIBERSORT (Chen et al., 2018; Zhang et al., 2022b) and CIBERSORT-ABS (Tamminga et al., 2020) were employed to assess immune infiltration levels. The association between risk scores and immune cells was analyzed using Spearman correlation analysis. The CIBERSORT algorithm was used to differentiate immune infiltration status between high-risk and low-risk groups. Additionally, single sample GSEA (ssGSEA) was employed to calculate immune function enrichment scores in UCEC patients. The Estimate algorithm was utilized to evaluate the composition of the tumor microenvironment (TME) for each UCEC sample, including the immune score, stromal score, and estimate cell infiltration (Yoshihara et al., 2013). To compare expression levels of 20 immune checkpoints with therapeutic potential between high-risk and low-risk groups, we referred to the work of Auslander et al. (2018).

We obtained two gene sets relevant to cancer-immune cycle and immunotherapy response from previously published studies (Mariathasan et al., 2018; Xu et al., 2018). The enrichment scores of these gene sets were calculated using Gene Set Variation Analysis (GSVA) to investigate their association with high-risk and low-risk groups (Hänzelmann et al., 2013). Correlation analysis between risk scores and these gene sets was performed using the R package ‘ggcor’. To predict the response to immune checkpoint inhibitors (ICIs), we utilized the Tumour Immune Dysfunction and Exclusion (TIDE) algorithm (Jiang et al., 2018). Validation of the risk model to predict immunotherapy effect was performed using the IMvigor210 cohort, for which full expression data and clinical information were obtained from (http://research-pub.Gene.com/imvigor210corebiologies/) (Mariathasan et al., 2018).

### 2.6 Somatic mutation analysis

Maftools is an R package that enables the analysis, visualization, and exploration of somatic mutation data in cancer research (Mayakonda et al., 2018). It has the ability to import mutation annotation files (MAFs) from various sources, including TCGA, and perform a variety of analyses such as mutation spectrum analysis, oncoplot visualization, and survival analysis based on mutational status. Moreover, it provides functions for extracting information on gene mutations, such as the frequency, type, and functional impact of mutations. In this study, we computed the tumor mutation burden (TMB) score for each UCEC patient and examined its correlation with the risk score. TMB score was determined by multiplying the quotient of total mutations and total covered bases by 106 (INVALID CITATIONa). We utilized the R package to perform Kaplan-Meier analysis to assess the prognostic value of TMB in UCEC patients. Additionally, we compared the distribution of microsatellite-stable (MSS), microsatellite instability-low (MSI-L), and microsatellite instability-high (MSI-H) tumor patients between the high-risk and low-risk groups.

### 2.7 Drug sensitivity

To investigate the therapeutic response of UCEC patients stratified into high-risk and low-risk groups based on their half-maximal inhibitory concentration (IC50) values retrieved from the Genomics of Cancer Drug Sensitivity (GDSC) database (https://www.cancerrxgene.org/), we employed the pRRophetic R package (Geeleher et al., 2014).

### 2.8 Cell culture

The human endometrial cancer cell lines Ishikawa, AN3CA and human endocervical epithelial cell line End1 were cultured in Dulbecco’s modified Eagle’s medium (DMEM; HyClone) supplemented with 10% fetal bovine serum (FBS; Hyclone), 100 U/L penicillin and 100 mg/L streptomycin (Thermo Fisher) at 37°C in 5% CO_2_.

### 2.9 qRT-PCR and IHC

The total RNA was extracted using the RNA Eazy Fast Tissue/Cell Kit (TIANGEN Biotech Co., Beijing) following the manufacturer’s instructions. The cDNA was synthesized using the FastKing RT Kit (TIANGEN Biotech Co., Beijing) according to the provided protocol. Real-time PCR was carried out using the SuperReal PreMix Plus (TIANGEN Biotech Co., Beijing) reagent and the StepOnePlus Real-Time PCR System. The PCR reaction was performed as follows: pre-denaturation at 95°C for 15 min, followed by 40 cycles of denaturation at 95°C for 10 s, annealing at 72°C for 20 s, and extension at 60°C for 20 s. The primer sequences used in the PCR reaction are listed in Table 1. Immunohistochemical analysis of sections from the HPA database (https://www.proteinatlas.org/).

**TABLE 1:** Primers used in qRT-PCR analysis.

Gene	Sequences (5′-3′)
H-GAPDH-F	CAA​TGA​CCC​CTT​CAT​TGA​CC
H-GAPDH-R	GAC​AAG​CTT​CCC​GTT​CTC​AG
H-FAM13C-F	CTT​GCC​TTG​CAA​TGC​CAT​GT
H-FAM13C-R	CTG​GAT​CTT​CGT​CAC​ACT​CTG​T
H-FAM110B-F	GAA​GGA​ATA​AGG​CGC​CCG​AC
H-FAM110B-R	CAG​CAG​GAA​ATT​GCT​CCC​ACA
H-FAM72A-F	TGG​GGT​CTG​ACA​TCA​ACG​A
H-FAM72A-R	AGT​GAA​GTC​CAC​TGC​GTT​CTC

### 2.10 Statistical analysis

The statistical analyses were performed using R software version 4.1.3. To compare the overall survival (OS) between the high- and low-risk groups, we utilized the Kaplan-Meier (KM) survival curves and log-rank test. The FFGs signature was constructed using the LASSO-Cox regression model, and its predictive performance was evaluated using time-dependent receiver operating characteristic (ROC) analysis. To assess the correlation between the risk score and immune cell infiltration, Spearman correlation analysis was employed. The proportion of tumor-infiltrating immune cells, immune checkpoints, and immune function between the two groups was compared using the Wilcox test. The qRT-PCR analyses were performed using GraphPad Prism Software (version 8.3.0). The results are presented as means ± standard deviation (SD) from three independent experiments, and statistical analysis was carried out using analysis of variance (ANOVA). The significance level was set at *p*-values <0.05 and false discovery rate (FDR) < 0.05.

## 3 Result

### 3.1 Identification of candidate FFGs

**FIGURE 1:**
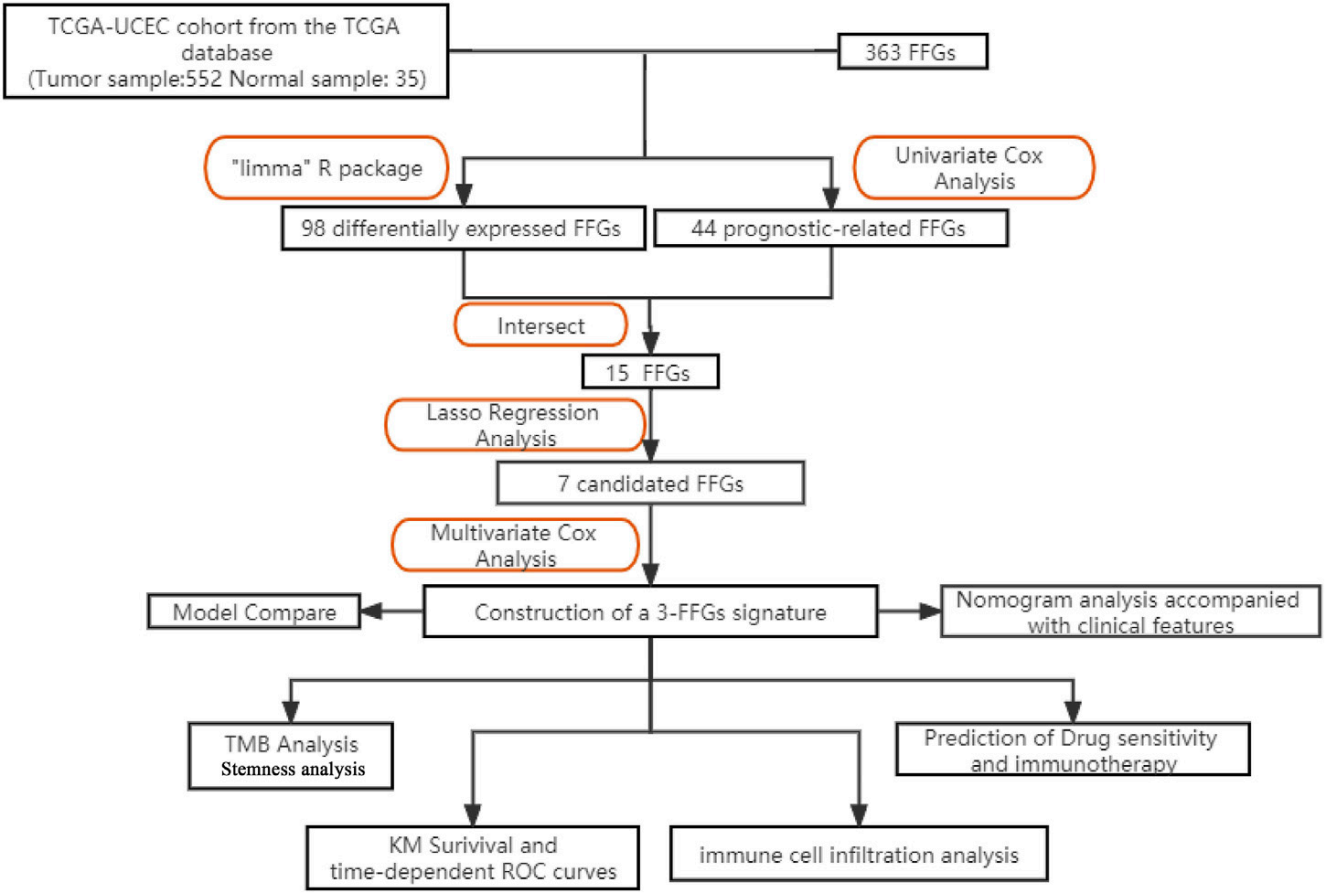
The diagram provides an overview of the primary design of the current investigation.

**FIGURE 2:**
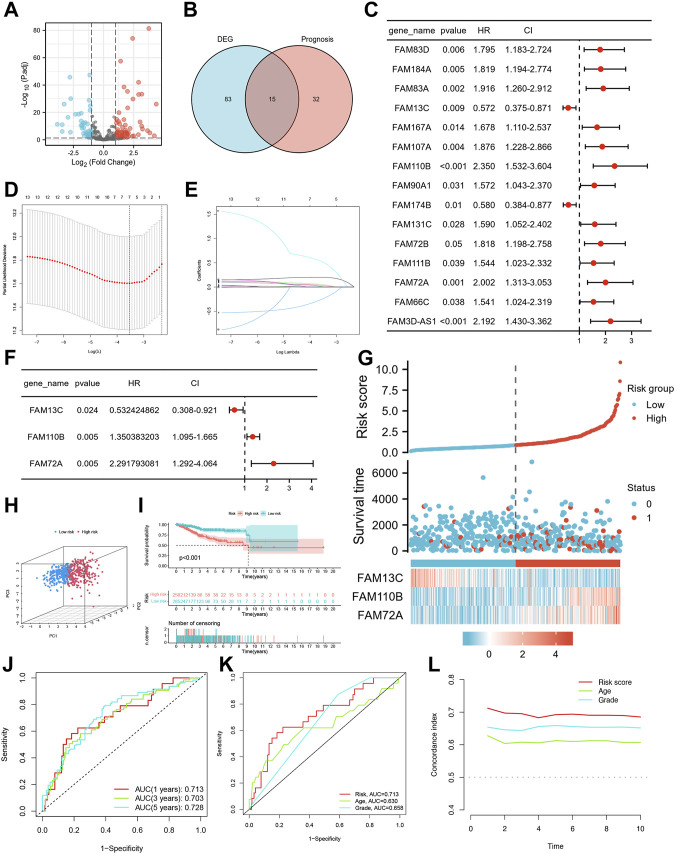
Identification of candidate FFGs and construction of prognostic signature. **(A)** Volcano map of 363 differentially expressed FAM family genes. **(B)** Venn diagram of the intersection of DE-FFGs and prognosis-related FFGs. **(C)** Prognosis of 15 FFGs in the entire cohort of UCEC was analyzed by univariate Cox regression model. **(D)** Ten‐time cross‐validation for tuning parameter selection in the LASSO model. **(E)** LASSO coefficient profiles. **(F)** Cox proportional risk regression model identified FAM13C, FAM110B and FAM72A as survival predictor signature. **(G)** Heatmap of risk factor in the test cohort. **(H)** PCA plot in the entire cohort. **(I)** K-M survival curve of **e**ndometrial cancer patients in the entire group. **(J)** Time-dependent ROC curves analysis. **(K)** Multi-index ROC analysis in the entire cohort. **(L)** Decision curve analysis.

The study design is depicted in Figure 1, which outlines the steps taken to identify a biomarker capable of predicting UCEC prognosis using a risk score model based on FAM family genes. We first analyzed the expression profiles of 363 FAM family genes in UCEC tumor tissues (n = 552) and paracancerous tissues (n = 35) using the “DESeq2” R package, resulting in 98 differentially expressed FAM family genes (DE-FFGs), of which 57 were upregulated and 41 were downregulated (|log2FC|>1, adjusted *p*-value <0.05) (Figure 2A; Supplementary Table S1). We then conducted a univariate Cox analysis using the “survival” R package and extracted 47 FFGs that were significantly associated with UCEC prognosis (*p* < 0.05) (Supplementary Table S2). By intersecting the 98 DE-FFGs with the 47 prognosis-related FFGs, we identified 15 differential expression FFGs, namely, FAM83D, FAM184A, FAM83A, FAM13C, FAM167A, FAM107A, FAM110B, FAM90A1, FAM174B, FAM131C, FAM72B, FAM111B, FAM72A, FAM66C, and FAM3D-AS1 (Figure 2B), which were significantly associated with overall survival (OS) in UCEC patients according to the univariate Cox analysis (Figure 2C).

### 3.2 Construction of FFGs prognosis signature with its predictive value

We conducted lasso regression analysis on 15 potential FFGs and identified 7 FFGs, including FAM184A, FAM83A, FAM13C, FAM167A, FAM110B, FAM90A1, and FAM72A (Figures 2D, E). Subsequently, a Cox proportional risk regression model was used to further narrow down the FFGs to 3, namely, FAM13C, FAM110B, and FAM72A, which had corresponding regression coefficients of −0.6303, 0.3004, and 0.8293 (Figure 2F). Next, we constructed a linear prediction model based on the 3 FFGs that were weighted by their regression coefficients through multivariate Cox analysis. We calculated the risk score for each patient using the formula: risk score = (−0.6303 × FAM13C expression level) + (0.3004 x FAM110B expression level) + (0.8293 x FAM72A expression level) for the entire cohort. Using the median cut-off point, we divided the patients into high-risk and low-risk groups, and observed that as the risk score increased, so did the mortality rate (Figure 2G). We employed PCA to visualize the risk distribution, and significant differences and a clear separation between high-risk and low-risk patients were observed (Figure 2H). Furthermore, Kaplan-Meier survival analysis showed that high-risk patients had a worse prognosis than low-risk patients (*p* < 0.001) (Figure 2I). We used time-dependent ROC curves to evaluate the model’s accuracy, and the AUC values for risk score 1, 3, and 5-year OS prediction were 0.713, 0.703, and 0.728, respectively, indicating high specificity and sensitivity (Figure 2J). The risk score for the three FFGs (AUC = 0.714) was a better predictor of UCEC prognosis than age and grade compared to common clinicopathological features (Figure 2K). This finding was consistent with our model having the highest C-index, indicating its superior clinical application based on the 3 FFGs (Figure 2L).

### 3.3 Validation of the FFGs prognostic model

**FIGURE 3:**
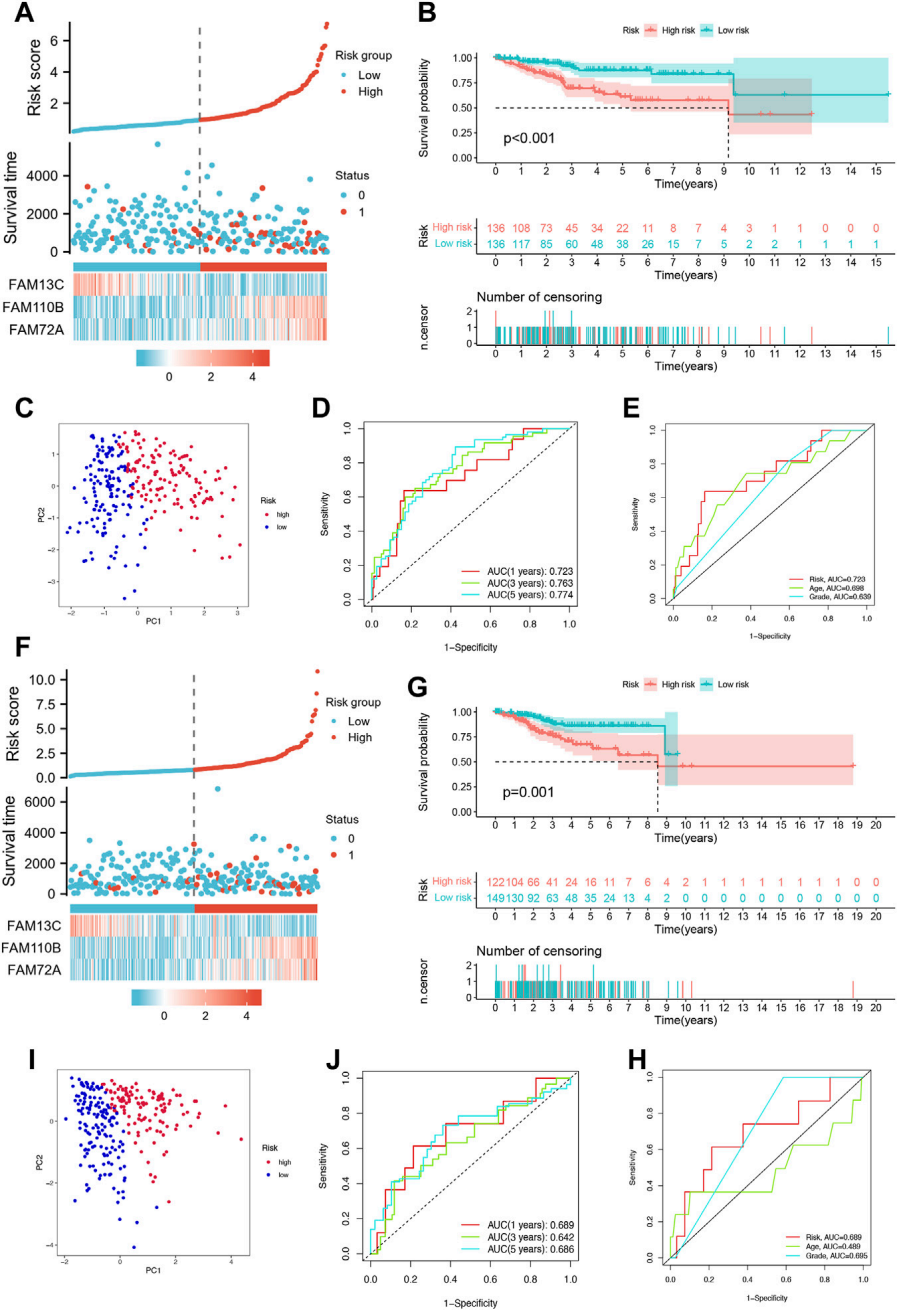
Validation of the prognosis signature for FFGs. **(A)** Heat map of risk factors in the train cohort. **(B)** K-M survival curve of UCEC patients in the train cohort. **(C)** PCA plot in the train cohort. **(D)** Time-dependent ROC curve of UCEC patients in the train cohort. **(E)** Multi-index ROC analysis in the train cohort. **(F)** Heatmap of risk factor in the test cohort. **(G)** K-M survival curve of UCEC patients in the test cohort. **(H)** PCA plot in the test cohort. **(I)** Time-dependent ROC curve of UCEC patients in the test cohort. **(J)** Multi-index ROC analysis in the test cohort.

To evaluate the prognostic accuracy of our model, we randomly divided the cohort of Uterine Corpus Endometrial Carcinoma (UCEC) patients into a training set (n = 272) and a testing set (n = 271). In the training set, we observed an increase in UCEC patient survival with increasing risk severity, as shown in Figure 3A. We validated our model by performing Kaplan-Meier analysis, which showed that high-risk patients had a worse prognosis than low-risk patients (*p* < 0.001), as illustrated in Figure 3B. Principal Component Analysis (PCA) revealed significant differences between low- and high-risk patients, which allowed for clear separation, as shown in Figure 3C. Furthermore, the area under the curve (AUC) of the time-dependent ROC curves for the training set demonstrated the model’s predictive power. Specifically, the 1-year AUC was 0.723, the 3-year AUC was 0.763, and the 5-year AUC was 0.774, as shown in Figure 3D.Our model also outperformed traditional clinicopathological features such as age and grade, as shown in Figure 3E, with an AUC of 0.723 for the three FFGs. In the testing set, we obtained similar results to the training set, where higher patient mortality was observed with increasing risk score (Figure 3F). Kaplan-Meier survival analysis showed that high-risk patients had worse prognoses than low-risk patients (*p* = 0.001), as shown in Figure 3G. PCA confirmed that low- and high-risk patients were significantly different and clearly separated (Figure 3H). Moreover, the time-dependent ROC curves for the testing set showed an AUC of 0.689 at 1 year, 0.642 at 3 years, and 0.686 at 5 years (Figure 3I). Our model again outperformed traditional clinicopathological features, with an AUC of 0.689 for the three FFGs, as shown in Figure 3J. In conclusion, our prognostic model demonstrated excellent performance, as evidenced by its ability to accurately predict the prognosis of UCEC patients based on three FFGs, outperforming traditional clinicopathological features.

### 3.4 Establishment of nomograms in combination with clinical characteristics

**FIGURE 4:**
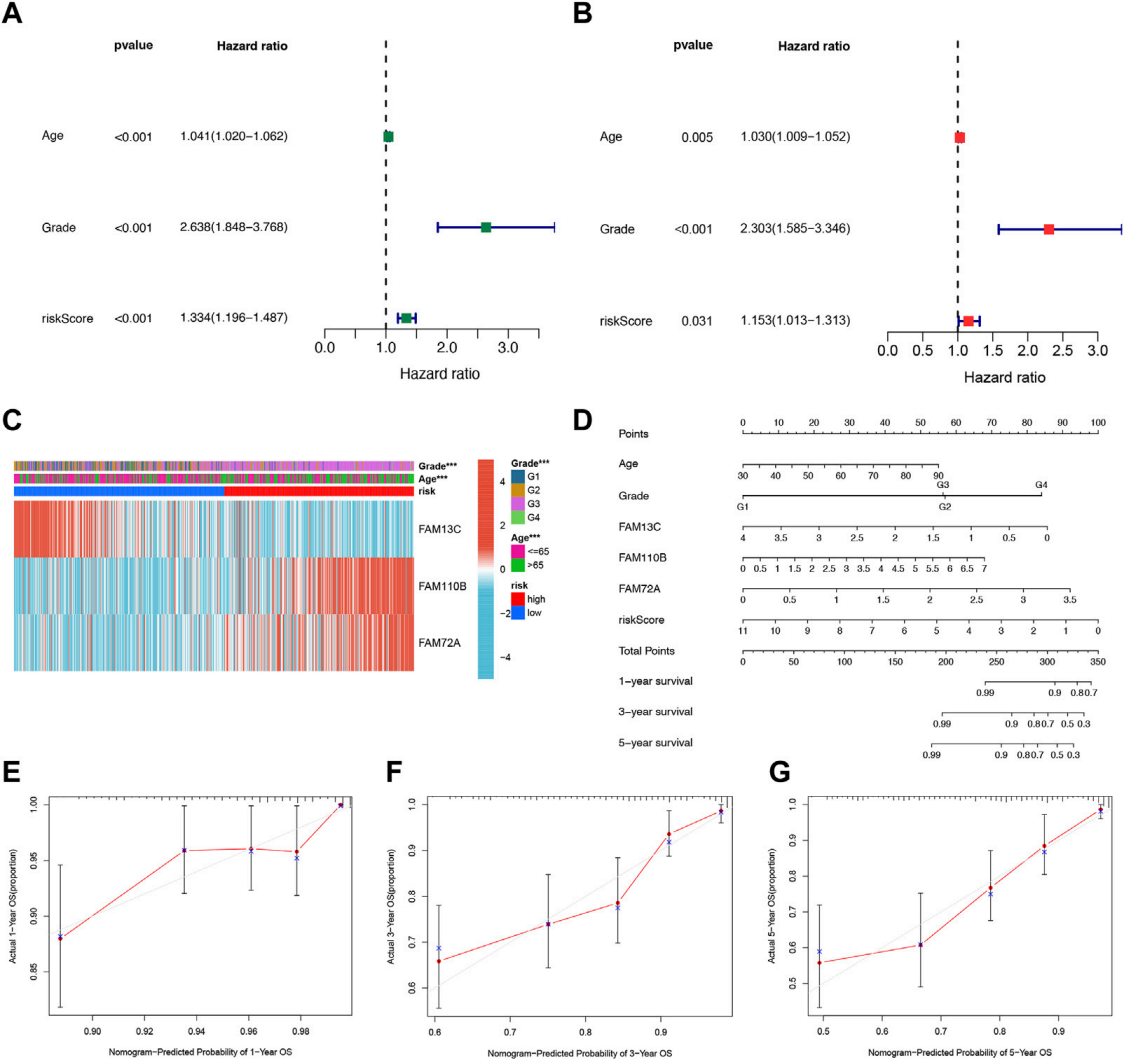
Independent prognostic analysis of risk scores and clinical parameters. Univariate **(A)** and multivariate **(B)** COX regression analysis of the signature and different clinical feature. **(C)** Heatmap for the 3 FFGs-based signature with clinicopathological manifestations. **(D)** Nomogram for predicting 1-year, 3-year, and 5-year OS of patients with UCEC. The calibration curve of the constructed nomogram of 1- year **(E)**, 3- year **(F)**, and 5-year **(G)** survival.

We further evaluated the potential clinical application of our prognostic risk model by conducting univariate and multivariate Cox analyses. These analyses aimed to determine whether the prognosis signature based on the three FFGs could serve as an independent prognostic factor for UCEC. Our univariate analysis revealed that age, grading, and risk score were significantly associated with UCEC patient prognosis (*p* < 0.001) (Figure 4A). Subsequently, we performed a multivariate analysis to adjust for age and grading, and found that the risk score remained an independent and reliable predictor of risk for the cohort (*p* = 0.031) (Figure 4B). To better understand the relationship between the three FAM genes identified in our prognostic risk model and the age, grade, and risk score of all UCEC samples from the TCGA, we generated a heat map (Figure 4C). To enhance the clinical utility and applicability of our constructed risk model, we developed a nomogram based on age, grade, FAM13C, FAM110B, FAM72A expression, and risk score. This nomogram predicted 1-year, 3-year, and 5-year survival probabilities for UCEC. Our model demonstrated that the risk score had the greatest impact on predicting overall survival, suggesting that the risk model based on the three genes, FAM13C, FAM110B, and FAM72A, was more effective in prognosticating UCEC (Figure 4D). Furthermore, calibration curves indicated that the predicted values were in satisfactory agreement with the observed values in terms of 1-year, 3-year, and 5-year OS probabilities (Figures 4E–G). In summary, our findings suggest that the constructed prognostic risk model based on the three FAM genes, FAM13C, FAM110B, and FAM72A, has potential clinical utility in predicting prognosis for UCEC patients. Our nomogram may serve as a valuable tool for clinicians to make informed decisions regarding patient management and treatment planning. Notably, our model demonstrates higher predictive accuracy compared to the conventional age and tumor grading system.

### 3.5 The FFGs signature performed better than others in prognostic prediction

**FIGURE 5:**
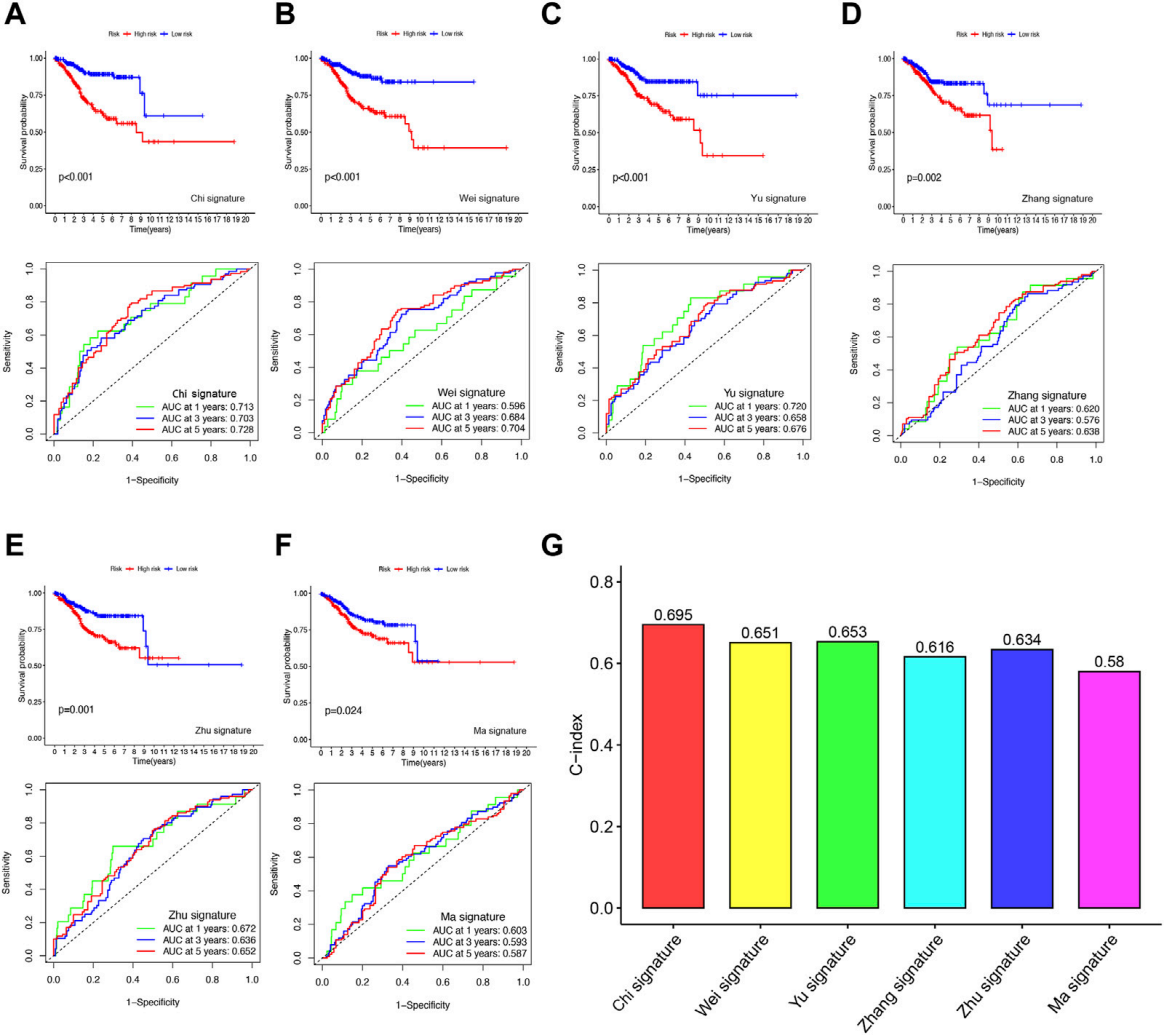
Comparison of the FFGs risk model with other models **(A)** KM curves and ROCs for FFGs signature. **(B–F)** KM curves and ROCs for risk models constructed by others. **(G)** C-indexes for six risk models.

To evaluate the predictive accuracy of our FFGs signature in UCEC, we compared it with five previously reported prognostic signatures, namely, the 4-gene signature by Huang et al. (2021), the 4-gene signature by Yu et al. (INVALID CITATIONb), the 2-gene signature by Liu et al. (2021a), the 3-gene signature by Liu et al. (2021b), and the 3-gene signature by Wang et al. (2020b). TTo enable a fair comparison, we used the same risk score calculation method for all UCEC samples in the TCGA database and transformed the scores based on the methods used in the five existing signatures. While all five signatures successfully distinguished UCEC patients into high- and low-risk groups with significantly different prognoses, our FFGs signature (Chi et al.) exhibited superior performance in time-dependent ROC curve analysis, as evidenced by higher AUC values for 1-year, 3-year, and 5-year survival (Figures 5A–F). Furthermore, our FFGs signature showed the highest C-index of 0.695 (Figure 5G), implying its superior predictive accuracy. Taken together, our findings indicate that our FFGs signature has high predictive accuracy in forecasting UCEC patient outcomes.

### 3.6 Differential expression and prognostic analysis of three FFGs in UCEC

We also evaluated the diagnostic potential of FAM13C, FAM110B, and FAM72A in distinguishing UCEC tissues from non-tumor tissues using ROC curves. All three genes demonstrated high AUC values, suggesting their potential as ideal biomarkers for this purpose (Supplementary Figure S1A). We further analyzed the relationship between the expression levels of these genes and clinicopathological characteristics of UCEC patients. Our findings revealed that FAM13C and FAM110B expression were significantly associated with age (*p* < 0.05), while FAM72A expression was not (Supplementary Figure S1B). Moreover, FAM13C expression decreased with increasing histological grading (*p* < 0.001), whereas FAM110B expression was significantly higher in G3 than in G1 and G2 (*p* < 0.001). FAM72A expression increased with increasing histological grading (*p* < 0.001) (Supplementary Figure S1C).

We performed survival analyses on the TCGA-UCEC dataset to investigate whether FAM13C, FAM110B, and FAM72A can serve as biomarkers in conjunction with other survival indicators. Our results indicated that low expression levels of FAM13C were significantly associated with poorer disease-specific survival (DSS) (*p* = 0.037) (Supplementary Figure S1D) and were strongly correlated with progression-free interval (PFI) (*p* = 0.033) (Supplementary Figure S1E). Conversely, high expression levels of FAM110B were closely linked to poorer DSS (*p* < 0.001) (Supplementary Figure S1F) and PFI (*p* = 0.001) (Supplementary Figure S1G). Similarly, high expression levels of FAM72A were strongly associated with poorer DSS (*p* < 0.001) (Supplementary Figure S1H) and PFI (*p* < 0.001) (Supplementary Figure S1I). These findings suggest that FAM13C, FAM110B, and FAM72A could serve as useful biomarkers in conjunction with other survival indicators for predicting clinical outcomes in UCEC patients.

### 3.7 FFGs risk score predicts TME and immune cell infiltration

**FIGURE 6:**
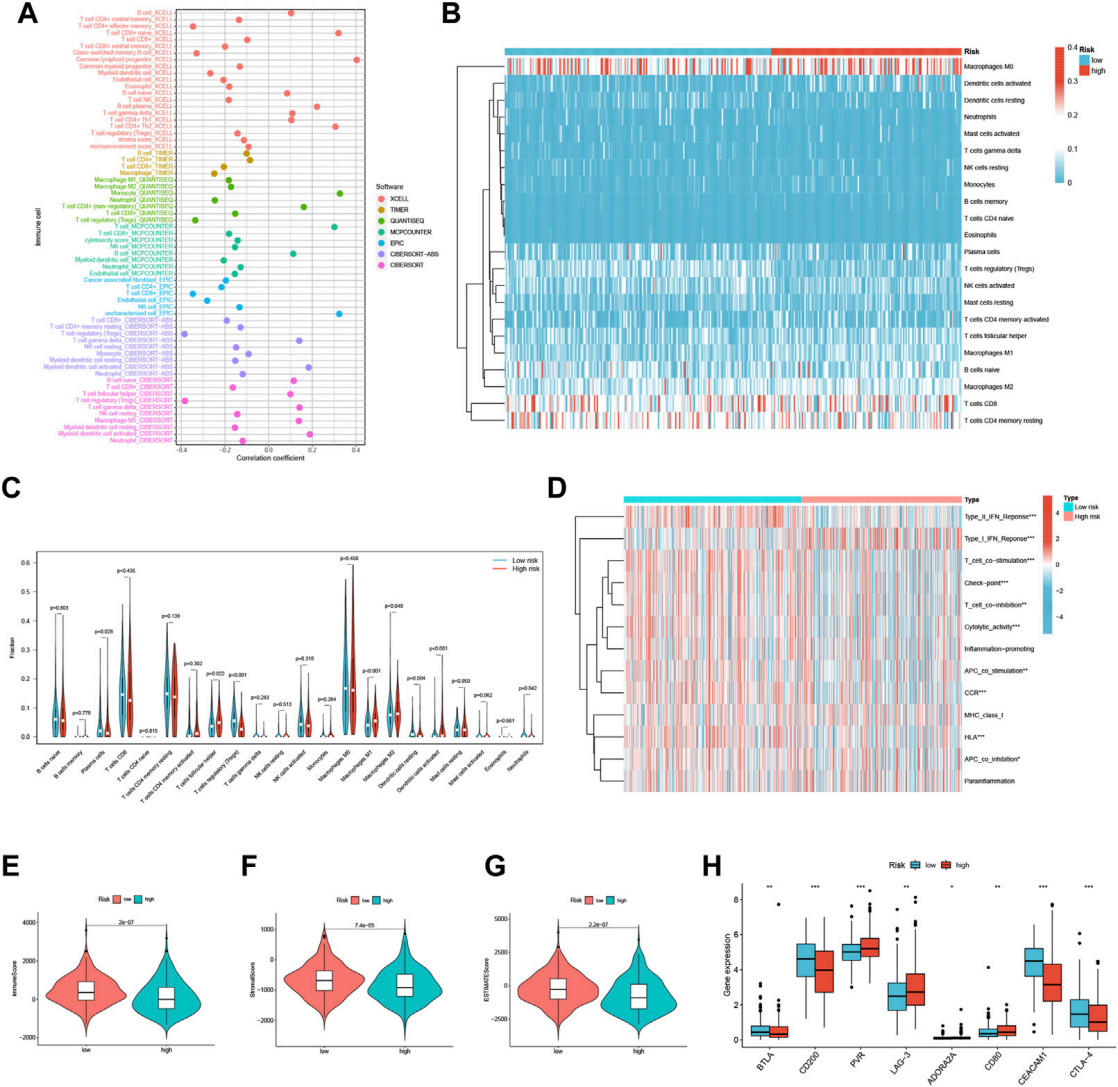
TME and immune cell infiltration in the two risk score groups. **(A)** Immune cell bubble plots of the risk groups. **(B)** Heat map showing the correlation between the 22 TICs. Correlation tests were performed with Pearson’s coefficient. **(C)** The ratio of immune cells between high-risk and low-risk groups. **(D)** Immune function and immune cell ssGSEA scores between high-risk and low-risk groups. **(E)** Immune score, **(F)** stromal score, **(G)** ESTIMATE score, **(H)** immune checkpoint between high-risk and low-risk groups *p < 0.05; **p < 0.01; ***p < 0.001.

We conducted a comprehensive investigation to explore the relationship between risk scores and the abundance of infiltrating immune cells in the context of endometrial cancer. To achieve this, we utilized a range of computational algorithms, including XCELL, TIMER, QUANTISEQ, MCPCOUNTER, CIBERSORT, CIBERSORT-ABS, and EPIC. Our findings demonstrate a significant inverse correlation between risk scores and Treg cells in XCELL, QUANTISEQ, CIBERSORT, and Cibersort-ABS, suggesting that the low-risk group may be associated with strong immunosuppression (Figure 6A). Further analysis of the distribution and correlation of 22 TICs in the TCGA-UCEC cohort using the CIBERSORT algorithm showed that most immune cells were negatively correlated with both high-risk and low-risk groups, except for macrophage M0, CD8^+^ T cells, and CD4^+^ T cells memory resting (Figure 6B). Notably, we observed higher levels of Plasma cells, Tregs, Dendritic cells resting, and Neutrophils in the low-risk group, while T cells follicular helper, macrophage M1, macrophage M2, and Dendritic cells activated were lower (Figure 6C). These findings suggest that specific immune cell types may be impacted by the three FFGs-related patterns, potentially influencing the response to immunotherapy.

We also analyzed the ssGSEA scores for immune cells and immune functions and discovered that nine immune functions, such as HLA and CCR, were more associated with the low-risk group, while only Type I IFN Response was more associated with the high-risk group (Figure 6D). In addition, we utilized the ESTIMATE algorithm to determine the TME composition of the UCEC samples and found that the immune score, Stromalscore, and ESTIMATE score were higher in the low-risk group compared to the high-risk group, indicating a higher overall immune level and immunogenicity of the tumor microenvironment in the low-risk group (Figures 6E–G). Finally, we examined the expression of immune checkpoints and identified four immune checkpoint genes significantly upregulated in the low-risk group (BTLA, CD200, CEACAM1, and CTLA-4), while four immune checkpoint genes were significantly upregulated in the high-risk group (PVR, LAG-3, ADORA2A, and CD80) (Figure 6H). Our results suggest that the risk score may provide valuable guidance for clinicians regarding the use of immune checkpoint-targeted drugs in endometrial cancer patients.

### 3.8 FFGs risk score predicts treatment response assessment

**FIGURE 7:**
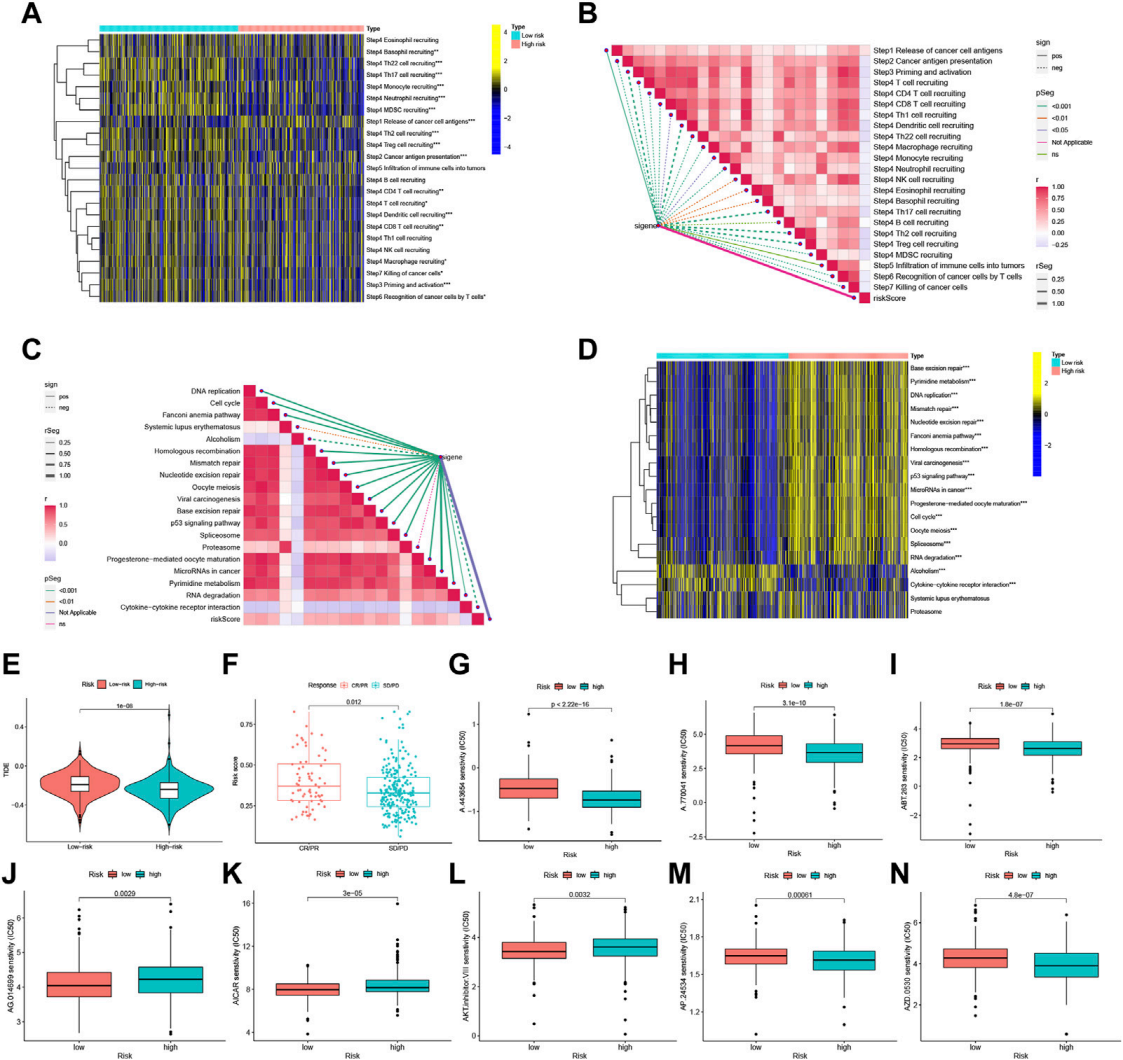
FFGs risk score predicts treatment response assessment. **(A)** Heat map of differences in the individual steps of the cancer-immune cycle between high and low risk groups. **(B)** Correlation of different risk scores with steps of the cancer-immune cycle. **(C)** Correlation of different risk scores with enrichment scores of immunotherapy prediction pathways. **(D)** Correlation between different risk scores and clinical response to immunotherapy. **(E)** Box-line graphs of TIDE scores in the high-risk *versus* low-risk groups in the TCGA UCEC cohort. **(F)** Correlation between risk scores and clinical response to cancer immunotherapy. Differences in IC50 of immunotherapy drugs by risk score **(G)** A.443654, **(H)** A.770041, **(I)** ABT.263, **(J)** AG.014699, **(K)** AICAR, **(L)** AKT. inhibitor, **(M)** AP.24534, **(N)** AZD.0530. PD, disease progression; SD, disease Stable; PR, partial response; CR, complete response. TIDE, Tumor Immune Dysfunction and Exclusion. *p < 0.05, **p < 0.01, ***p < 0.001.

Assessment of the tumor immune cycle is critical for understanding the role of immune modulators, such as the chemokine system (Chen and Mellman, 2013; Xu et al., 2018). In the low-risk group, upregulation of activity was observed for most steps in the cycle, including cancer cell antigen expression (step 2), initiation and activation (step 3), and transport of immune cells to the tumor (step 4) (CD4^+^ T-cell recruitment, CD8^+^ T-cell recruitment, T cell recruitment, dendritic cell recruitment, basophil recruitment, Th22 cell recruitment, macrophage recruitment, Th2 cell recruitment, Treg cell recruitment, monocyte recruitment, neutrophil recruitment, and Th17 recruitment). In contrast, cancer cell antigen release (step 1) activity was decreased, and T cell recognition of cancer cells (step 6) was increased (Figures 7A, B). Correlation analysis between different risk scores and the predicted ICB response signatures revealed that the majority of patients in the low-risk group were negatively correlated with the enrichment scores of ICB-related positive signatures. However, the low-risk group was only positively correlated with Cytokine-cytokine receptor interaction and had no significant relationship with RNA degradation, Systemic lupus erythematosus, and Proteasome (Figures 7C, D).

Using the Tumor Immune Dysfunction and Exclusion (TIDE) algorithm to predict the likelihood of the immunotherapy risk model, TIDE was significantly higher in the low-risk group than in the high-risk group (P < 1e-08) (Figure 7E), indicating that patients in the low-risk group are less likely to benefit from ICI (immune checkpoint inhibitor) therapy due to the higher likelihood of immune evasion. FFG expression was also significantly higher in patients who responded to treatment (CR or PR) compared to those who showed stable or progressive disease (*p* = 0.012) (Figure 7F).

Furthermore, the low-risk group exhibited a higher IC50 for five immunotherapeutic agents applied to UCEC treatment, including A.443654 (*p* < 2.22e-16), A.770041 (*p* = 3.1e-10), ABT.263 (*p* = 1.8e-07), AP.24534 (*p* = 0.00061), and AZD.0530 (*p* = 4.8e-07). In contrast, we also identified three other chemical or targeted drugs [AG.014699 (*p* = 0.0029), AICAR (P = 3e-05), AKT. inhibitor.VIII (*p* = 0.0032)] that exhibited a lower IC50 in the low-risk group (Figures 7G–N). Based on these findings, risk score analysis may aid in further investigation of immunotherapy response in UCEC patients and may enhance the precision of drug therapy.

### 3.9 Comparison of somatic mutation between low-risk and high-risk groups

**FIGURE 8:**
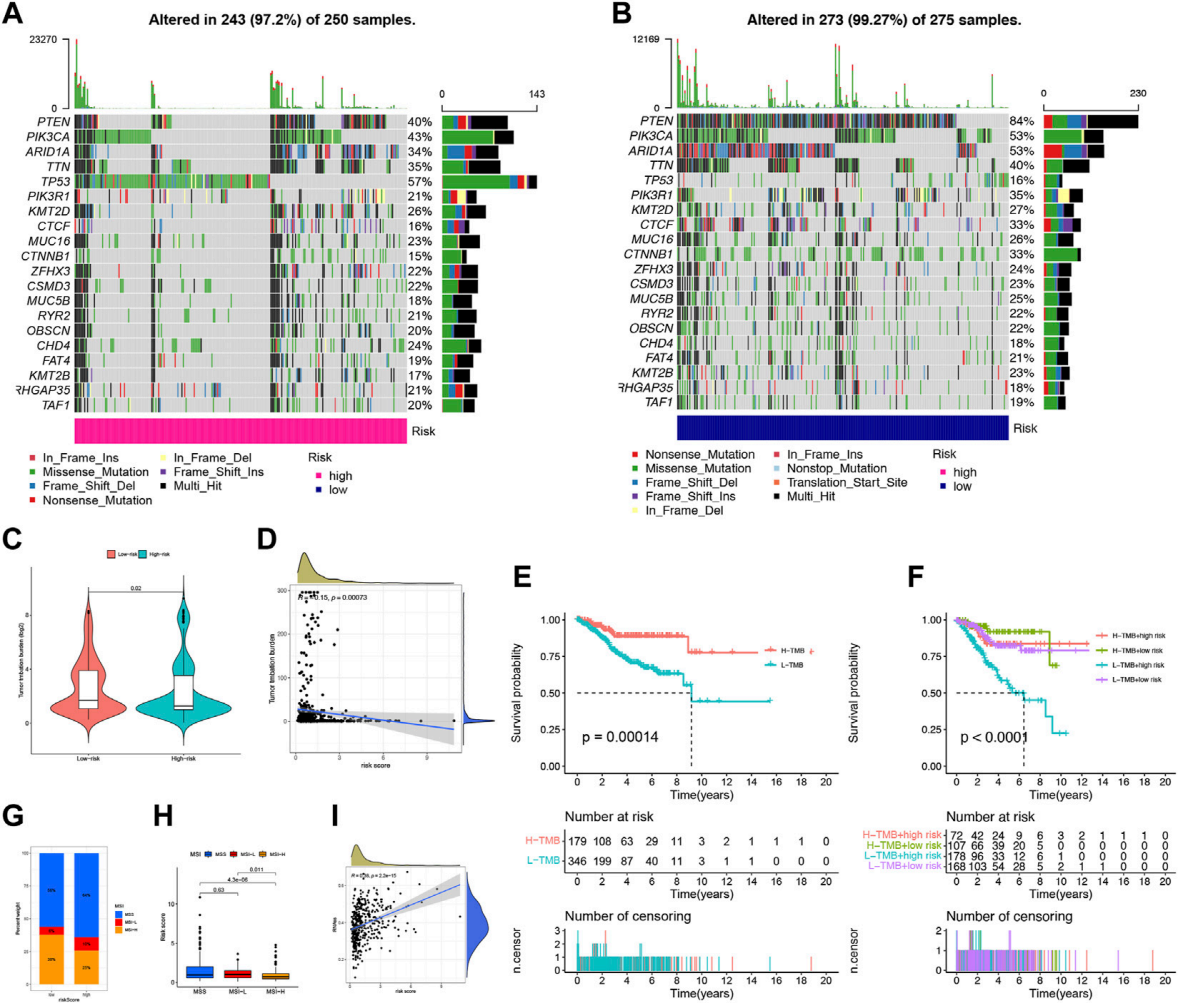
Landscape of mutation profiles in UCEC samples. **(A,B)** The hub-mutated markers in both groups. **(C)** The TMB between high-risk and low-risk patients. **(D)** Correlation between the TMB and risk score. **(E)** Kaplan-Meier analysis showing the relationship between the level of TMB and clinical outcome (*p* = 0.00014). **(F)** Effects of distinct TMB in different Risks on the survival probability (*p* < 0.001). **(G)** The proportion of different MSI in the high-risk and low-risk score groups. **(H)** The relationship between the MS and risk score. **(I)** The correlation relationship between FFGs expression and CSC index.

We conducted an analysis of somatic mutations in UCEC patients to distinguish between high-risk and low-risk groups. The most commonly mutated genes in high-risk patients were TP53 (57%), PIK3CA (43%), and PTEN (40%), while PTEN (84%), PIK3CA (53%), and ARID1A (53%) were the most frequently mutated genes in low-risk patients (Figures 8A, B). Patients with a higher tumor mutation burden (TMB) may benefit from immunotherapy due to their increased neoantigen load (Snyder et al., 2014). We computed TMB for both risk groups and observed a significantly higher TMB in the low-risk group than in the high-risk group (*p* = 0.02), indicating that patients in the low-risk group may be more responsive to immunotherapy (Figure 8C). Furthermore, we found that the risk score was negatively correlated with TMB (Figure 8D). We assessed TMB’s prognostic potential for UCEC patients and observed that patients with low TMB values had worse survival rates than those with high TMB values (*p* = 0.00014) (Figure 8E) (Samstein et al., 2019). When combining TMB with risk scores, we found that low-risk patients with high TMB had the best prognosis, whereas high-risk patients with low TMB had the worst prognosis (*p* < 0.0001) (Figure 8F). MSI is another predictive biomarker for cancer immunotherapy. We assessed the immune prediction of MSI for high-risk and low-risk groups of patients and observed that MSI-H had the lowest risk score, indicating that low-risk patients with MSI-H had lower immune risk and better immune prediction (*p* = 0.011) (Figures 8G, H). Additionally, we examined the relationship between FFG-Score and CSC indices (RNAss) to evaluate potential correlations between FFG-Score and CSCs. We found a positive correlation between FFG-Score and the CSC index (R = 0.38, *p* = 2.2e-15), indicating that a higher FFG-Score was associated with more pronounced stem cell properties and less cellular differentiation (Figure 8I).

### 3.10 Validation of the built model by RT-qPCR and IHC

**FIGURE 9:**
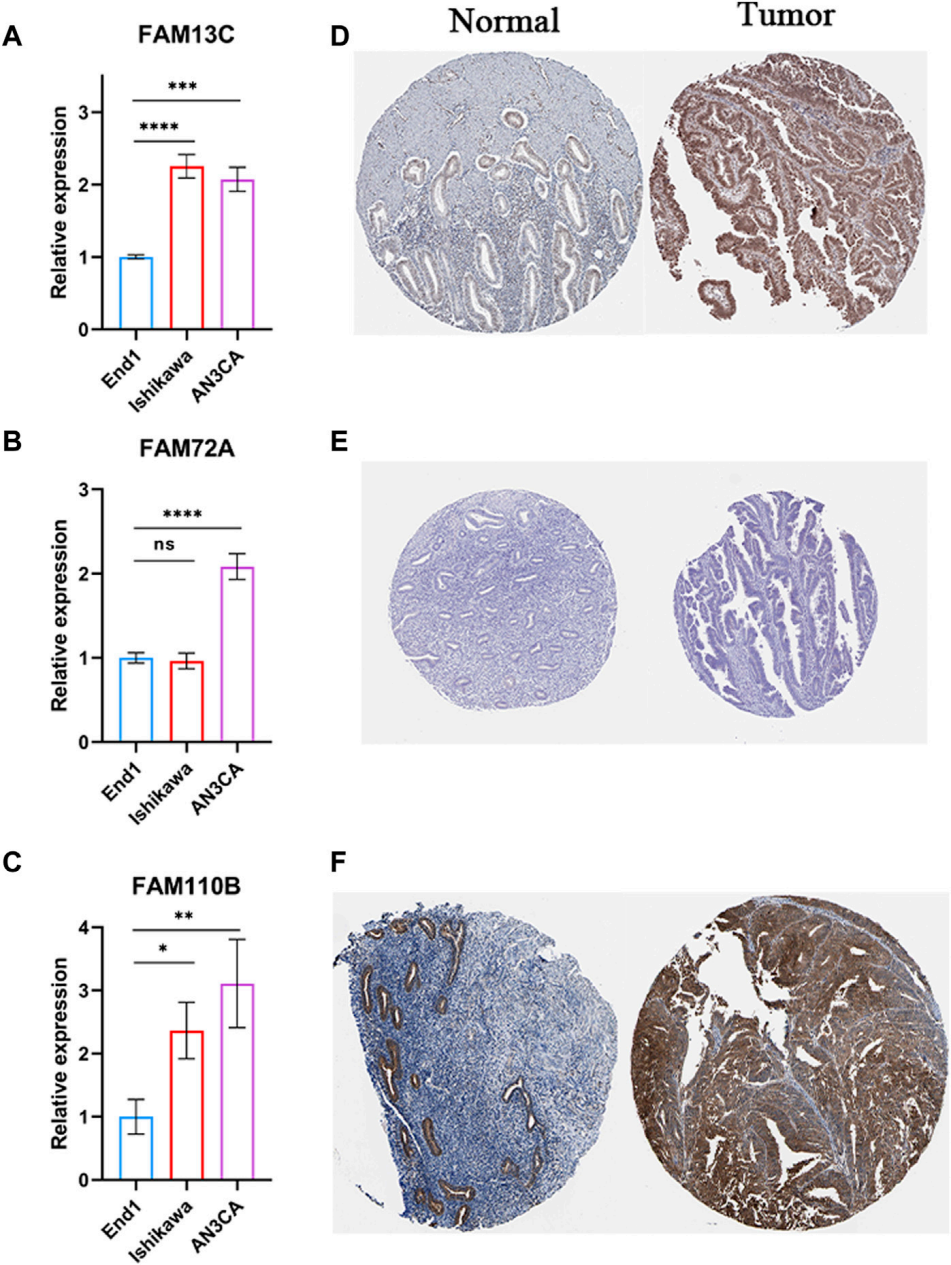
Results of RT-qPCR and IHC experiments on 3-FFGs. RT-qPCR result of **(A)** FAM13C, **(B)** FAM72A, **(C)** FAM110B. IHC result of **(D)** FAM13C, **(E)** FAM72A, **(F)** FAM110B.*p < 0.05; **p < 0.01; ***p < 0.001; ****p < 0.0001.

We performed experiments to investigate the expression of 3-FFGs in participating endometrial cancer cells and normal endocervical cells. Our results showed that the expression levels of FAM13C, FAM72A and FAM11B were all significantly upregulated in endometrial cancer cells compared to normal endocervical cells (Figures 9A–C). In addition, the results of immunohistochemical analysis showed that FAM13C and FAM110B had higher protein expression in endometrial cancer tissues, while FAM72A showed no difference (Figures 9D–F). From the above results, we speculate that the expression status of these FGGs may be complexly associated with the development of endometrium.

## 4 Discussion

Endometrial carcinoma (UCEC) is a prevalent gynecologic malignancy that is associated with unfavorable prognosis and low survival rates (Brooks et al., 2019; Raffone et al., 2019). However, conventional categorization of UCEC has limitations in capturing the tumor’s diverse characteristics (Liu et al., 2021b). Single-gene-based prognosis prediction for UCEC is challenging, given the numerous factors that can affect gene expression. Instead, a combined model consisting of multiple relevant genes can provide greater precision in predicting prognosis and is crucial for personalized treatment (Hu et al., 2021a). Despite growing evidence of the FAM family genes’ significant impact on the tumor microenvironment, no comprehensive analysis of the FFGs in UCEC exists. To address this gap, we analyzed mRNA expression data from the TCGA-UCEC dataset to identify significant prognostic genes and develop a multi-biomarker prognostic model based on the FAM family genes. Our study findings indicate that FFGs-based signatures could be utilized for risk stratification, prognosis prediction, and evaluation of immunotherapy efficacy in UCEC, providing valuable resources for personalized treatment.

In this study, we developed a prognostic model for FFGs based on the TCGA-UCEC dataset. We employed lasso regression and COX risk regression analyses to select three genes (FAM13C, FAM110B, and FAM72A) for our model from a pool of 363 FFGs. The resulting FFGs signature was found to be an independent prognostic factor for UCEC and effectively stratified UCEC patients into two prognostic subgroups. Moreover, our analysis demonstrated the strong predictive performance of the FFGs signature, as confirmed by ROC and calibration curve analyses. To increase the clinical applicability of our model, we constructed a nomogram that integrated clinical factors and risk scores. Our FFGs-based model, which includes fewer genes than other UCEC prediction models, exhibited superior predictive performance and may serve as a valuable tool for evaluating prognosis in UCEC patients.

The FAM13C gene encodes a protein whose function and cellular localization have not been fully characterized. However, its structural domain suggests its potential involvement in intracellular signaling pathways relevant to cancer (Cohen et al., 2004). Burdelski et al. have shown that overexpression of FAM13C is a strong and independent prognostic factor in prostate cancer (Burdelski et al., 2017). FAM110B, a member of the FAM110 gene family, is primarily located in centrosomes and is involved in microtubule nucleation and organization in tissues. It can influence the progression of the G1 phase of the cell cycle when overexpressed and has been found to limit proliferation and invasion of non-small cell lung cancer by inhibiting Wnt/β-linked protein signaling (Hauge et al., 2007; Xie et al., 2020). In prostate cancer cells, FAM110B knockdown reduces cell viability and induces apoptosis, suggesting its potential as a therapeutic target. Furthermore, FAM110B has been implicated in the regulation of tumor cell surface antigen presentation, which may impact immune evasion by tumor cells (Vainio et al., 2012; Colak et al., 2013). Notably, the therapeutic potential of targeting FAM110B has been demonstrated not only in pancreatic and colon cancers, but also in several other cancer types, including non-small cell lung cancer and prostate cancer (Xi and Zhang, 2018; Wang et al., 2020a). FAM72A, also known as Ugene, is a recently discovered neuronal protein that has been implicated in tumorigenesis in multiple tissues (Pramanik et al., 2015). It accelerates the G1/S phase transition in the cell cycle and promotes cancer cell survival (Wang et al., 2011; Heese, 2013). FAM72A may affect the balance of mutagenic DNA repair and increase the likelihood of cells acquiring mutations, potentially contributing to tumor development (Guo et al., 2008; Renganathan et al., 2021). Additionally, FAM72A has been identified as a new prognostic factor for patients with hepatocellular carcinoma (Zhang et al., 2021; Gao et al., 2022). Although the role of FAM family genes, such as FAM13C, FAM110B, and FAM72A, in UCEC remains unclear, their relevance to prognosis and the tumor immune microenvironment in UCEC patients warrants further investigation. FAM72A is a newly discovered gene, and its research in the field of oncology is still in its infancy. However, FAM72A expression has been found to be significantly upregulated in UCEC and correlated with the occurrence, development and prognosis of UCEC. Therefore, future studies can further investigate the mechanism of FAM72A in UCEC, explore the feasibility of FAM72A as a prognostic marker and explore the potential value of FAM72A as a therapeutic target, and provide a theoretical basis for the development of new therapeutic approaches targeting FAM72A.

The tumor microenvironment (TME) is a complex system comprising various cells, growth factors, and signaling molecules that regulate tumor progression and immune escape (Sahoo et al., 2018; McCoach and Bivona, 2019). Here, we aimed to investigate the relationship between the FAM gene family and the TME in uterine corpus endometrial carcinoma (UCEC). We conducted a systematic analysis of the risk and high-risk groups in terms of immune infiltration and found that patients in the low-risk group had higher levels of plasma cells and CD8^+^ T cells, indicating a better immune response against tumor cells (Kondratiev et al., 2004). T-cell co-stimulation is a hierarchical process that is crucial for the formation of an effective immune response, while Tregs are a subpopulation of CD4^+^ T cells that play a critical role in tumor immune escape and angiogenesis (Facciabene et al., 2012; Tanaka and Sakaguchi, 2017; Chao and Savage, 2018). T-cell co-stimulation is a hierarchical process that is crucial for the formation of an effective immune response, while Tregs are a subpopulation of CD4^+^ T cells that play a critical role in tumor immune escape and angiogenesis (Hu et al., 2021b).

In addition, we observed the upregulation of suppressive immune checkpoint molecules, including CTLA-4, BTLA, CD200, and CEACAM1, in the low-risk group. Previous studies have reported that high levels of CTLA-4 expression are associated with a better prognosis in UCEC patients (Spranger et al., 2013; Liu et al., 2020). Blockade of BTLA immune checkpoint molecules has been shown to improve lymphocyte function and enhance the efficacy of anti-PD-1 monoclonal antibody therapy in UCEC (Panda et al., 2020; Demerlé et al., 2021). CD200 blockade limits pancreatic tumor growth and enhances the efficacy of PD-1 blockade in preclinical animal models (Choueiry et al., 2020). CEACAM1 has also been identified as a potential target for immunotherapy of tumors due to its ability to suppress the immune activity of TIL (Turcu et al., 2016). Thus, these immune checkpoint inhibitors may be promising targets for combination therapy with anti-PD-1 monoclonal antibodies in low-risk UCEC patients to enhance the immunotherapeutic effect.

The tumor microenvironment (TME) plays a crucial role in tumor progression and response to treatment, and immune cell infiltration and immune checkpoint inhibitors have been recognized as key factors in the antitumor immune response. Recently, tumor mutational burden (TMB) has also emerged as a crucial factor in immunotherapy. TMB is calculated as the number of somatic gene coding errors, base substitutions, gene insertions or deletions detected per million bases in the tumor genome, excluding germline mutations. These somatic mutations can result in the production of new or altered proteins/peptides, which can be recognized as foreign by the immune system, leading to an anti-tumor immune response (INVALID CITATIONb). Studies have demonstrated that in PD-L1 end-selected or PD-L1-positive populations, the response to PD-1/PD-L1 inhibitor therapy is positively associated with higher TMB (Lawrence et al., 2013; Rizvi et al., 2015). TMB has predictive value for immunotherapy in various tumors and has been shown to be a better predictor of efficacy than PD-L1 expression (Carbone et al., 2017; Wang et al., 2019). However, assessing TMB in routine clinical practice is challenging due to high sequencing costs and long turnaround times. In this study, we found that FFGs are closely correlated with TMB, indirectly reflecting TME status and providing valuable information for immunotherapy outcomes. We observed a significant negative correlation between FFGs and TMB, and low-risk patients had significantly higher TMB than high-risk patients, indicating that the low-risk group is more likely to benefit from immunotherapy. Combining TMB with risk scores can effectively predict the prognosis of UCEC patients, providing a possible clinical practice reference for guiding immunotherapy in UCEC.

Although our study provides important insights for prognostic assessment and treatment selection in UCEC patients, it has several limitations that must be acknowledged. Firstly, due to its retrospective nature, our findings require validation in prospective studies. Secondly, the precise molecular mechanisms underlying the prognostic impact of FAM family genes in UCEC patients remains to be fully elucidated and should be further explored through *ex vivo* experiments. Thirdly, we attempted to identify external validation cohorts for our findings, but we were unable to identify appropriate datasets. Therefore, it is crucial to establish an independent cohort of patients to confirm our results. Lastly, the TCGA-UCEC cohort is predominantly composed of white and black patients, with only a small number of Asians represented. Future studies should aim to include more diverse populations to ensure the generalizability of our findings.

## 5 Conclusion

In summary, the study has developed a prognostic model using FAM family genes that can accurately predict the prognosis of UCEC patients. Moreover, the FFGs signature could provide valuable information on the immune status of patients and identify potential immunotherapy options for UCEC treatment. The development of this prognostic model and FFGs signature could lead to personalized treatment approaches for UCEC patients, ultimately improving their outcomes.

## Data Availability

The original contributions presented in the study are included in the article/Supplementary Material, further inquiries can be directed to the corresponding authors.

## Supplementary material

SUPPLEMENTARY FIGURE S1 Differential prognostic analysis of the three FFGs in UCEC. **(A)** ROC curves showing the efficiency of FAM13C, FAM110B and FAM72A expression levels in differentiating UCEC tissues from non-tumor tissues. Association of FAM13C, FAM110B and FAM72A gene expression with clinical characteristics including **(B)** age and **(C)** histological grade. Kaplan-Meier survival analysis of different FAM13C, FAM110B and FAM72A expression status in UCEC from TCGA dataset, including **(D)** FAM13C disease-specific survival, **(E)** FAM110B disease-specific survival, **(F)** FAM72A disease-specific survival, **(G)** FAM13C progression-free interval, **(H)** FAM110B progression-free interval and **(I)** FAM72A progression-free interval. **p* < 0.05; ***p* < 0.01; ****p* < 0.001.

SUPPLEMENTARY TABLE S1 Expression level profiles of FAM family genes.

SUPPLEMENTARY TABLE S2 Prognostic levels of FAM family genes.
