# Supplementary figures and images for: FAM family gene prediction model reveals heterogeneity, stemness and immune microenvironment of UCEC
Source: Front Mol Biosci. 2023 May 19;10:1200335. doi: 10.3389/fmolb.2023.1200335 (PMC10235772; doi:10.3389/fmolb.2023.1200335)

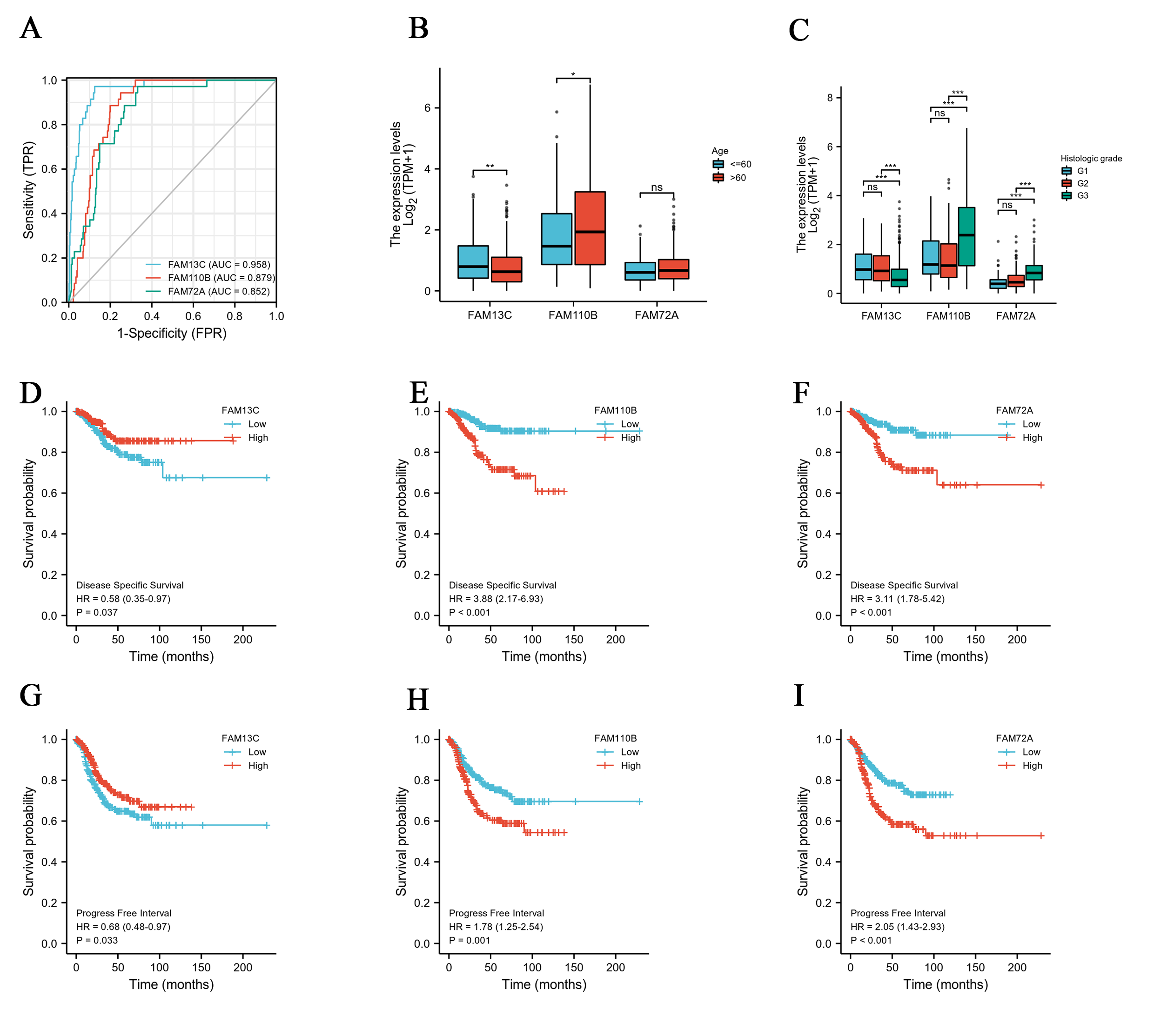

Supplement: Supplementary file 2 [file Image1.TIF]
